# Open Capsular Repair with Dermal allograft and tarsal tunnel release in the treatment of Tarsal Tunnel Syndrome in a Young Collegiate Athlete: A Case Report

**DOI:** 10.1177/2050313X241271773

**Published:** 2024-08-13

**Authors:** Bryan N Ubanwa, Chimobi C Emukah, David M Heath, Marie T Chapentier, Robert O Cone, Kenneth Kenneth-Nwosa, Katherine C Bartush

**Affiliations:** 1 University of Texas Health Science Center San Antonio Joe and Teresa Lozano Long School of Medicine, San Antonio, TX, USA; 2 Department of Orthopaedic Surgery, University of Texas Health Science Center San Antonio, San Antonio, TX, USA; 3 Department of Radiology, University of Texas Health Science Center San Antonio, San Antonio, TX, USA

**Keywords:** Orthopedics/Rehabilitation/Occupational therapy, Sports medicine, Tarsal tunnel, Foot, Ankle

## Abstract

Tarsal tunnel syndrome is an entrapment neuropathy of the posterior tibial nerve beneath the flexor retinaculum that can be precipitated by either intrinsic or extrinsic factors. We report a unique case of a posterior medial ankle joint capsular defect with localized fluid extravasation between the flexor digitorum longus and flexor hallucis longus leading to symptoms consistent with tarsal tunnel syndrome in a collegiate tennis player. This patient is a 19-year-old female with no past medical history who presented with symptoms consistent with tarsal tunnel syndrome. After confirmation with magnetic resonance imaging, the patient underwent capsular reconstruction with dermal allograft in combination with a tarsal tunnel release. The patient had improvement in pain and recovery of paresthesia 3 months postoperatively. At the latest follow-up of 1 year postoperatively, the patient has not had a recurrence of symptoms and has returned to the same level of competitive play. Many different causes of tarsal tunnel syndrome are described in the literature, but to our knowledge, there is no current literature that describes a defect in the tibiotalar joint capsule as a cause of tarsal tunnel syndrome.

## Introduction

Tarsal tunnel syndrome (TTS) tends to be more common in athletes who are subjected to prolonged weight-bearing periods with intense physical activity.^1,2^ Tarsal tunnel syndrome can occur from both extrinsic and intrinsic causes including tight-fitting footwear, valgus hindfoot malalignment, or ganglion cysts. TTS has been reported to occur in athletes involved in strenuous sporting activities, especially in those with intense loading of the ankle joint such as sprinting and jumping.^3^ Anatomically, TTS is due to a compression neuropathy of the posterior tibial nerve or its branches, the lateral and medial plantar nerve, under the medial flexor retinaculum.^4,5^ TTS is usually made clinically. The ankle joint, also known as the talocrural or tibiotalar joint, is a large synovial joint formed by the articulation of the tibia, fibula, and talus and is surrounded by a relatively weak capsule.^6,7^ Inflammation or abnormalities of the ankle joint located adjacent to the tarsal tunnel can compress the tunnel and impinge on its contents, causing symptomatic changes to the medial and plantar foot.

## Case report

### Pre-procedure

A 19-year-old female Division 1 collegiate tennis player with no past medical history presented to the clinic with 1 year of right ankle pain. Though the exact number of years the athlete played tennis is unknown, the ability to participate in Division 1 tennis suggests she likely has extensive experience and background in the sport. The patient experienced minimal symptoms during normal activities of daily living; however, she reported experiencing pain during prolonged walking and active participation in tennis, particularly during movements such as side-to-side shuffling. The patient described a shooting, sharp pain down the medial aspect of her right ankle. She endorsed numbness and tingling along the medial plantar aspect of the first metatarsal. On examination, there was no obvious deformity to the right ankle. There was a positive Tinel’s sign present over the tarsal tunnel.^8^ She had been previously evaluated by a physician who diagnosed her with TTS, for which she received a single corticosteroid injection to the area and was prescribed physical therapy. Unfortunately, she had little improvement from these measures; therefore, advanced imaging and a nerve conduction study were ordered. Ultrasound imaging of the tarsal tunnel was not conducted.

**Figure 1.**
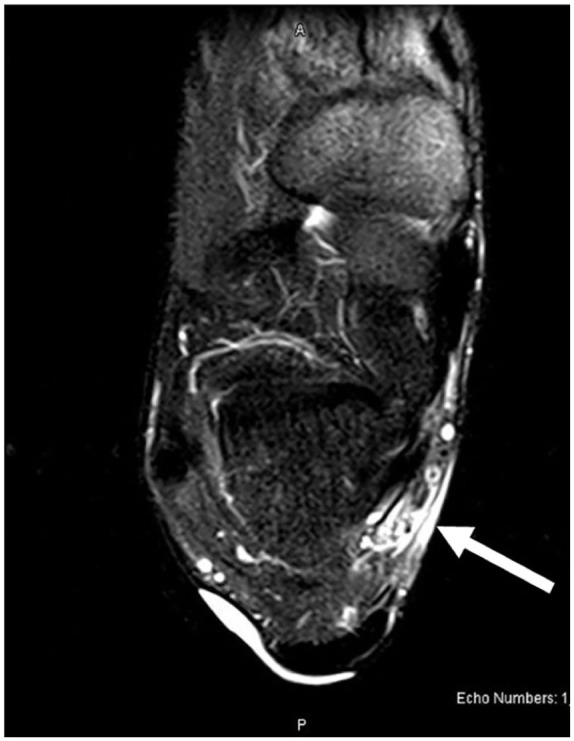
Magnetic resonance imaging: axial view.

**Figure 2.**
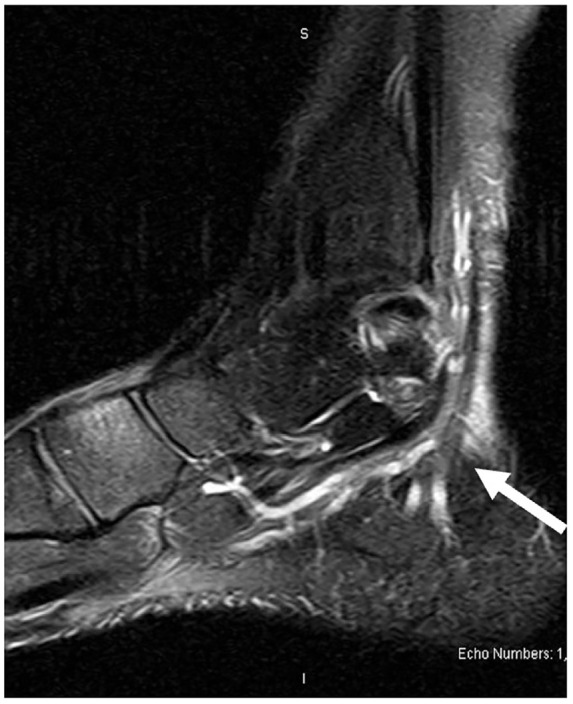
Magnetic resonance imaging: sagittal view.

**Figure 3.**
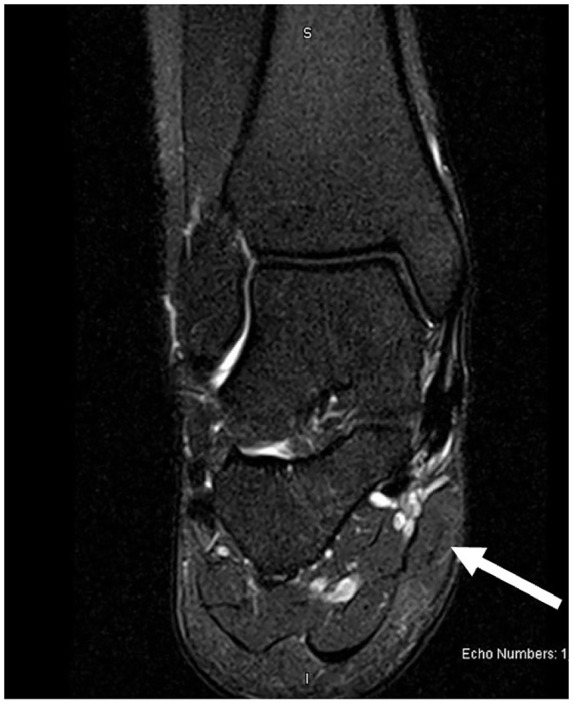
Magnetic resonance imaging: coronal view.

Magnetic resonance imaging (MRI) demonstrated nonspecific soft tissue edema and subcutaneous fluid collection along the posteromedial aspect of the ankle joint extending to the posterior ankle capsule at the level of the talus into the tarsal tunnel (Figures 1–3). There were no discrete solid or cystic masses. Nerve conduction studies results were not consistent with TTS.

It is difficult to determine whether there was a fluid collection at the time of the nerve conduction study, but it is presumed that there was not enough to induce a change in the study results, as it was normal.

**Figure 4.**
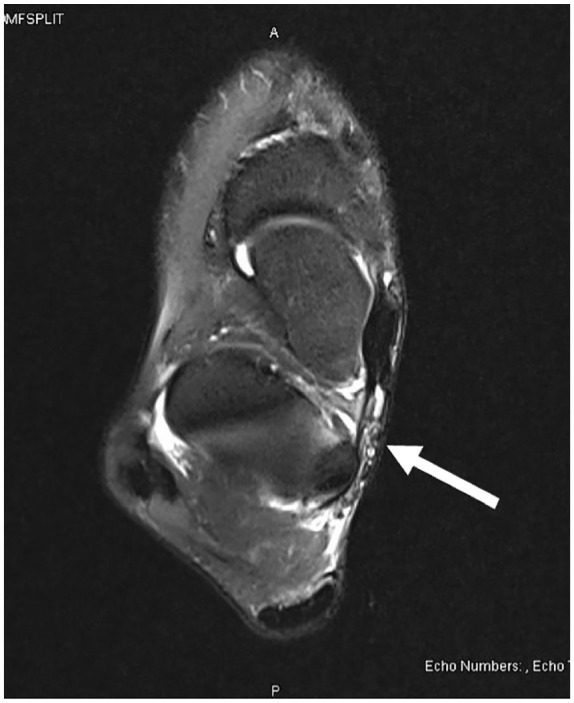
Magnetic resonance arthrogram: axial view.

**Figure 5.**
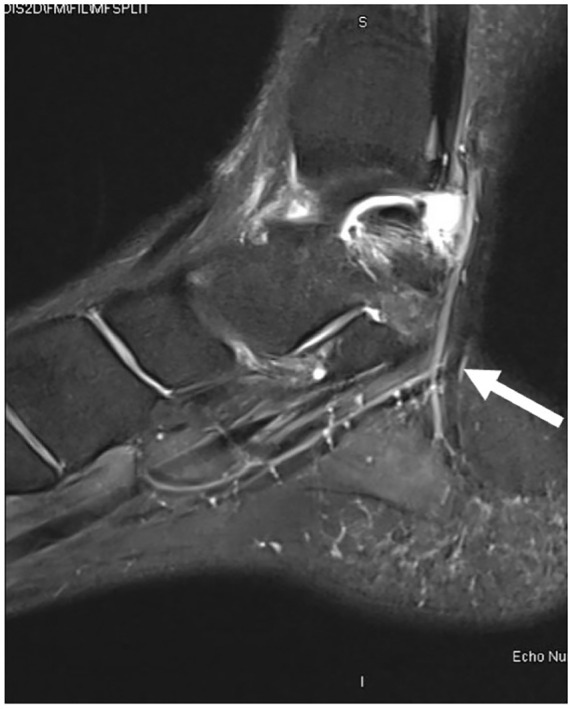
Magnetic resonance arthrogram: sagittal view.

**Figure 6.**
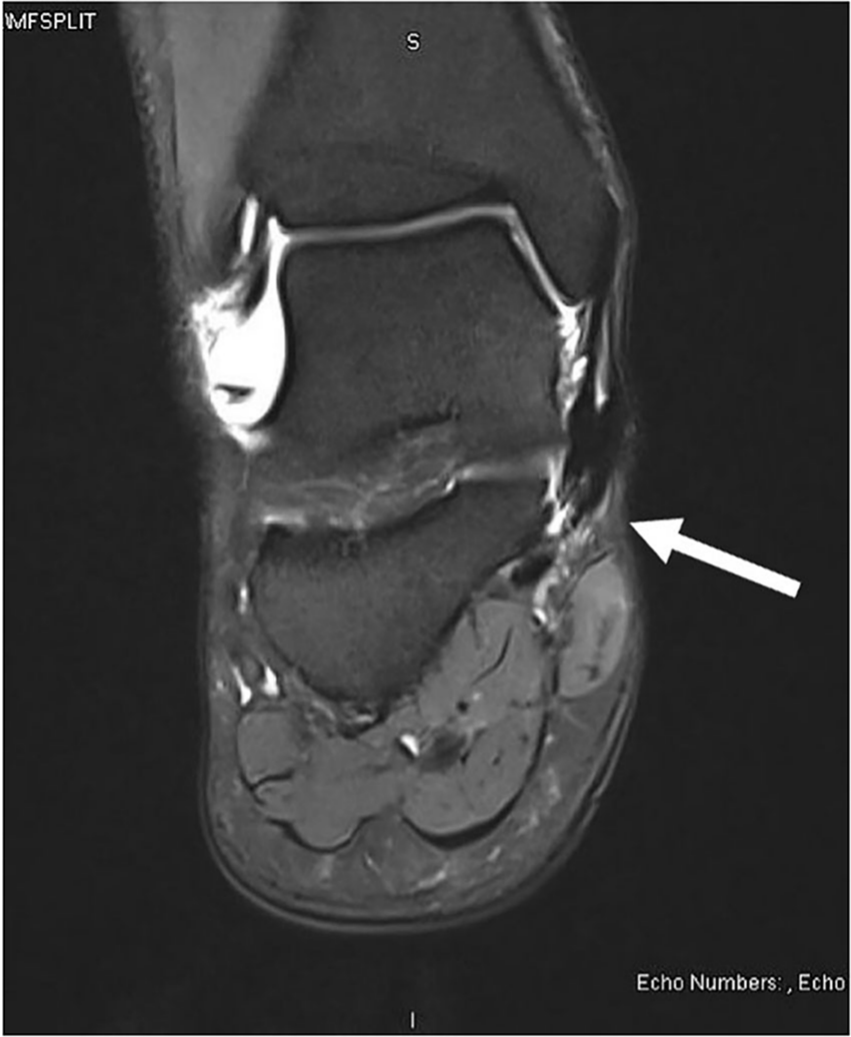
Magnetic resonance arthrogram: coronal view.

Upon presentation, the MRI was discussed with the reading radiologist who expressed his concern for a posteromedial ankle joint capsular defect. It was decided to obtain a magnetic resonance arthrogram (MRA) of the right ankle for further evaluation. During the MRA, fluoroscopic guided injection of a combination of radiopaque solution and an anesthetic solution was performed. Immediately following the injection, the patient had diminished (>70%) pain in the right ankle with ambulation and decreased pain response with manual pressure over the posteromedial ankle. Results of the MRA revealed extravasation of intra-articular contrast along the posterior medial aspect of the ankle joint extending along the margin of the posterior capsule between the flexor digitorum longus (FDL) and flexor hallucis longus (FHL) tendons (Figures 4–6). The finding confirmed a defect in the medial ankle joint capsule. After discussion, the patient elected to undergo a right ankle open capsule repair and tarsal tunnel release.

### Procedure

**Figure 7.**
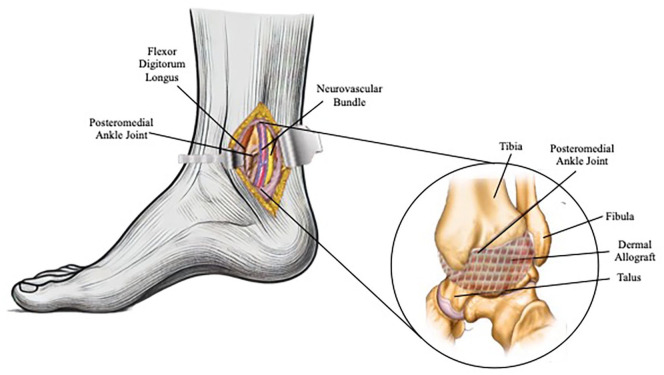
Illustration of the intraoperative capsular repair.

A posteromedial approach was made between the medial malleolus and the Achilles tendon. Figure 7 illustrates the capsular reconstruction with dermal allograft on the right ankle using a posteromedial approach, clearly depicting key anatomical structures surrounding the talocrural joint. The fascia overlying the tarsal tunnel was released to aid in the mobilization of the flexor tendons. The fascia over the FHL muscle belly was incised. The interval between the neurovascular bundle and FDL was used to identify the most medial aspect of the posterior capsular defect. Dermal allograft was then laid over the defect and sewn in a running fashion with 3-0 Fiberwire. The native ankle capsule was then laid over and tacked to the periosteum for augmentation of the repair. The patient was placed into a well-padded splint and was instructed to remain non-weight bearing for 2 weeks.

### Postoperative

At 3 months, the patient had decreased medial ankle pain but endorsed stiffness. Gentle range of motion training was prescribed to the patient as she transitioned to a short-controlled ankle movement boot. At the 6-month postoperative follow-up, the patient reported progressive improvement and decreased pain overall. At 1 year postoperatively, she had resolution of all symptoms. She was able to return to the same level of competitive play with occasional soreness when walking for periods longer than an hour. No images were obtained during subsequent postoperative visits, following the patient’s notable symptom relief.

## Discussion

TTS presents a diagnostic challenge due to its diverse etiologies, often involving compression or irritation of the tibial nerve within the tarsal tunnel. While literature extensively documents various causes of TTS, including anatomical variations, trauma, and systemic conditions, our case presents a novel finding: a defect in the tibiotalar joint capsule as a potential causative factor.

Currently, there are no published reports documenting a defect in the tibiotalar joint accompanied by secondary extravasation of synovial fluid as a causative factor in TTS. Although not described in the literature, inferences can be made regarding the etiology of the defect. Connective tissue disorders have been implicated in instances of hypermobility or laxity, predisposing individuals to microtrauma and subsequent capsuloligamentous damage.^2^ While the patient did not report significant trauma to the ankle, the etiology of the defect likely developed gradually over time, possibly due to microtrauma. We hypothesize that the issue is unlikely to stem from a congenital defect, given the adult onset of symptoms. However, although the patient had no history of diagnosed connective tissue disorder, it cannot be completely ruled out.

Nerve conduction studies are commonplace in the work-up and diagnoses of compressive neuropathies, although they do not always yield diagnostic results.^6^ Typically, TTS is diagnosed clinically utilizing a thorough history and physical examination, supplemented by nerve conduction studies and advanced imaging for additional diagnostic insight and anatomical etiology, respectively. Notably, in our case, the nerve conduction study results were negative. However, considering the transient nature of symptoms during strenuous activities, it is plausible that there was increased extravasation of synovial fluid acutely during times of exertion, leading to acute and symptomatic compression of the neural components within the tarsal tunnel. This acute compression scenario contrasts with chronic compression, which would more likely manifest observable changes in nerve conduction studies.

MRI is currently considered the best modality for delineation of the contents of the tarsal tunnel.^4,9^ Nariai et al.^7^ suggested that the optimal MRI technique for assessing the tarsal tunnel is the axial T1-weighted and axial fat-suppressed T2-weighted fat suppression sequence with thin slice sequences. While these sequences were utilized in our work-up, they did not allow us to identify the capsular defect present with our patient. The addition of the arthrogram did provide visualization of a capsular defect while also providing diagnostic information with intra-articular anesthetic briefly alleviating the patient’s pain.

Dermal allograft has been shown to have tissue integration properties and cellular incorporation.^7,10^ The use of dermal allograft is currently not described in the literature for tibiotalar joint capsule defects. Its use has mostly been described in the setting of superior capsular reconstructions for chronic, massive irreparable rotator cuff tears, but it has many other uses.^11^ Its biomechanical properties lent well to its application in this setting allowing for a robust repair of the capsule.

## Conclusion

In conclusion, this case illustrates a rare cause of intrinsic tibial nerve neuritis within the tarsal tunnel. A posteromedial ankle capsular defect with local intra-articular fluid extravasation leading to symptoms of TTS is not currently reported in the literature. Our case demonstrates that both magnetic resonance arthrography and a diagnostic intra-articular tibiotalar joint injection of an anesthetic away from the site of the defect may have utility in the diagnosis of TTS. We recommend that physicians with clinical suspicion collaborate with a skilled musculoskeletal radiologist, to aid in the diagnosis of such capsular defects.
